# Applying a Human-Centered Design to Develop a Patient Prioritization Tool for a Pediatric Emergency Department: Detailed Case Study of First Iterations

**DOI:** 10.2196/18427

**Published:** 2020-09-04

**Authors:** Jessica Schiro, Sylvia Pelayo, Alain Martinot, François Dubos, Marie-Catherine Beuscart-Zéphir, Romaric Marcilly

**Affiliations:** 1 Inserm, CIC-IT 1403/Evalab, F-59000 Lille France; 2 Univ Lille, CHU Lille, ULR 2694 - METRICS : Évaluation des technologies de santé et des pratiques médicales, F-59000 Lille France; 3 Paediatric Emergency Unit & Infectious Diseases, CHU Lille Lille France

**Keywords:** emergency department, triage systems, ergonomics, design, human-centered design, patients

## Abstract

**Background:**

Overcrowding in the emergency departments has become an increasingly significant problem. Patient triage strategies are acknowledged to help clinicians manage patient flow and reduce patients’ waiting time. However, electronic patient triage systems are not developed so that they comply with clinicians’ workflow.

**Objective:**

This case study presents the development of a patient prioritization tool (PPT) and of the related patient prioritization algorithm (PPA) for a pediatric emergency department (PED), relying on a human-centered design process.

**Methods:**

We followed a human-centered design process, wherein we (1) performed a work system analysis through observations and interviews in an academic hospital’s PED; (2) deduced design specifications; (3) designed a mock PPT and the related PPA; and (4) performed user testing to assess the intuitiveness of the icons, the effectiveness in communicating patient priority, the fit between the prioritization model implemented and the participants’ prioritization rules, and the participants’ satisfaction.

**Results:**

The workflow analysis identified that the PPT interface should meet the needs of physicians and nurses, represent the stages of patient care, and contain patient information such as waiting time, test status (eg, prescribed, in progress), age, and a suggestion for prioritization. The mock-up developed gives the status of patients progressing through the PED; a strip represents the patient and the patient’s characteristics, including a delay indicator that compares the patient’s waiting time to the average waiting time of patients with a comparable reason for emergency. User tests revealed issues with icon intuitiveness, information gaps, and possible refinements in the prioritization algorithm.

**Conclusions:**

The results of the user tests have led to modifications to improve the usability and usefulness of the PPT and its PPA. We discuss the value of integrating human factors into the design process for a PPT for PED. The PPT/PPA has been developed and installed in Lille University Hospital's PED. Studies are carried out to evaluate the use and impact of this tool on clinicians’ situation awareness and prioritization-related cognitive load, prioritization of patients, waiting time, and patients’ experience.

## Introduction

### Background

Emergency department (ED) overcrowding occurs when demand for emergency services exceeds the capacities to provide care [1-4]. Overcrowding has been shown to increase waiting times and, as a consequence, delay time-sensitive treatments and procedures for serious conditions, which in turn increases patient mortality and morbidity [5,6]. In 2004, the United Kingdom’s National Health Service introduced indicators to ensure patients are seen, admitted, and discharged within 4 hours of presentation to the ED [7]. Those indicators led to the development of specialized call centers, dedicated emergency units, mobile emergency medical teams [8], acute medical units [9], and new organizational protocols [10]. In the meantime, other strategies have been shown to improve patient flow and reduce waiting time [11,12], for example, having hospitalists manage beds [13], having nurses support patient movement [14], having physicians conduct early evaluation and manage patient flow [15], or performing patient registration at the bedside [16].

Triage of patients at their arrival is a long-established strategy to identify patients with critical conditions [17]. As soon as patients arrive in the ED, the severity of their condition is assessed and their treatments are prioritized accordingly [18,19]. This task is usually performed by triage nurses but is more efficient when performed by a senior physician [20] or by a physician and a nurse [21]. In this task, clinicians may use paper-based [22] or electronic triage systems [23-25].

Despite the weak evidence supporting the effectiveness of triage, this strategy is acknowledged to decrease waiting time [12] and to be a determinant of health care system performance [26]. However, the data used by today’s electronic patient triage systems must often be entered manually by clinicians; this is problematic when the ED is overcrowded and therefore limits the system’s usage and potential positive impact. Moreover, sorting algorithms implemented in the electronic triage systems are not based on actual strategies employed by the clinicians [24,25]. Therefore, there is a risk that those systems conflict with clinicians’ workflow and disturb their work.

### Study Context

The pediatric emergency department (PED) of Lille University Hospital has a capacity of approximately 30,000 patients per year. A total of 10 doctors and 8 nurses (plus residents and trainees) work in the department to take care of the patients. The department is currently equipped with ResUrgences (Berger-Levrault), a patient management software that tracks patients from their arrival in the PED through discharge. ResUrgences is independent of the hospital's electronic health records but is interconnected with the laboratory information system and the picture-archiving and communication system from which it receives notifications when results are available.

The PED’s clinicians enter the patient's data (eg, name, age, reason for admission, triage decision) and their own observations in ResUrgences. The patient record is progressively completed as the patient moves through the care process. However, ResUrgences does not prioritize the patients or organize their care accordingly, so clinicians must mentally compare the status of different patients and determine which one should be managed first. The Optimum project aims to develop and install in the PED a patient prioritization tool (PPT) as an extension to ResUrgences that does not require clinicians to enter additional information, but which enables them to have an accurate awareness of the waiting situation of patients and suggests to them which patient they should see next based on their current prioritization strategies. The information provided by the PPT should assist clinicians in prioritizing patients, thereby helping to decrease their cognitive load and optimize patient management in real time. Ultimately, using this tool could contribute to reduced waiting time, especially the waiting time for critical patients and for time-sensitive treatments and procedures.

Poor design of health technologies can ruin their expected benefits [27,28]. In addition, design problems are a serious problem for hospitals around the world, contributing to clinician burnout and impacting patient care [29,30]. Methods of the human-centered design process, a design process in which usability and users of the technology are the focus of attention at all design stages [31], contribute to the development of health technologies that correspond to the real needs of end users, respect users’ workflow, and reduce risk of use errors [32-36]. Thus, applying these methods to design a technology helps to reduce the risk of technology rejection on the one hand and to ensure that systems are more effective and efficient on the other [37-39].

**Figure 1:**
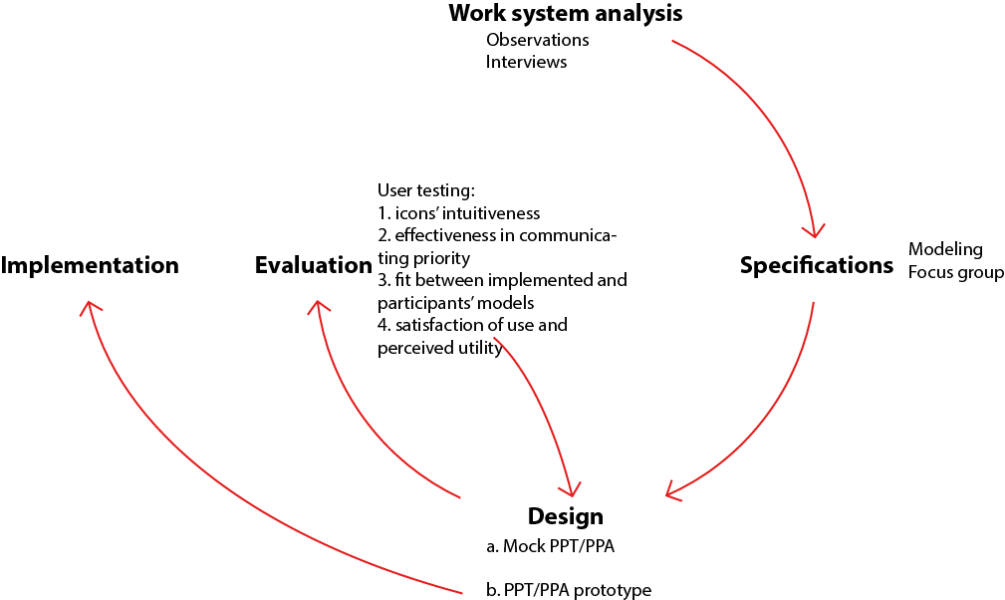
Representation of the human-centered design process applied during the study. First, a work system analysis was performed through observations and interviews. Then specifications were developed using modeling and a focus group session. A mock PPT/PPA was designed and then evaluated by user testing. Results from the evaluation helped improve the usability of the PPT/PPA prototype before implementation. PPA: patient prioritization algorithm; PPT: patient prioritization tool.

For the developed PPT to align with clinicians’ workflow and needs, the PPT was developed using a human-centered design process [31]. A work system analysis was performed and specifications were defined; then a mock tool was developed and underwent a usability evaluation (Figure 1). This case study presents the development of a PPT and of the patient prioritization algorithm (PPA) it relies on to show how to apply human-centered design methods to the design of triaging systems.

## Methods

### Work System Analysis and the Tool’s Functional Specifications and Design

The work system analysis had two main objectives. First, it aimed to identify the needs of clinicians and the constraints that shape their work. This required an in-depth understanding of the organization of the PED and how clinicians manage the flow of patients and prioritize them in overcrowded conditions. Second, it aimed to learn to what extent it was possible to use the data entered in ResUrgences to feed the PPT and PPA. This required knowing whether the data entered in the software accurately represented the actual state of patient care (ie, verifying that the data were entered quickly enough to track each patient’s progress through the various steps in care).

The data were first collected by structured observations performed by a human factors specialist using an observation grid built with iCoda (Studiocode). The observation unit consisted of the actions taken and the prioritization decisions made by the clinicians so that we could map the care process in detail and understand the prioritization decisions in depth. On a voluntary basis, 4 physicians and 4 registered nurses from the PED were individually shadowed during busy periods of the day (10 AM to 2 PM and 4 PM to 8 PM) over a 3-week period in February 2014 until the observations no longer provided new information.

Each action taken by clinicians was characterized in the observation grid as a communication with other clinicians, patients, or relatives; an interaction with documents or technologies (including ResUrgences); a move; an examination; a care; or an intervention. For each action observed, the grid made it possible to collect the step concerned in the care process; the action sequence in which the action took place; the profile of the clinician (eg, physician, registered nurse); the location; the type of information gathered, exchanged, or entered/written down (particularly prescriptions for care or procedures); etc. The data collected were time stamped so that the time interval between occurrence and data entry could be measured to know whether events were documented in ResUrgences in a timely manner.

In addition, clinicians were interviewed whenever the workload eased. They were asked to state and explain their reasons for patient prioritization, define the information on which their decisions were based, and state how and where they had collected this information. Furthermore, the same clinicians were formally interviewed at the end of their work shift with a focus on the data used to determine which patient should be taken care of first and how patients are prioritized. These interviews were audiorecorded and transcribed.

Data collected from the work system analysis were modeled through Unified Modeling Language diagrams [40] in order to highlight, for each step of the care process, interactions between clinicians, their usage of ResUrgences (eg, data consulted, data entered), and the data used to advance the process.

Clinicians' explanations of how they prioritize patients were analyzed qualitatively to extract common implicit and explicit patient-sorting rules that clinicians apply, the contexts in which these rules are applied, and the information used to make these prioritization decisions. Sorting rules were modeled using decision trees. Patient-sorting rules were combined and integrated into a PPA. All models and decision trees were validated by the clinicians observed and interviewed.

The information needs to be met by the PPT were deduced from the work model and the prioritization rules applied by clinicians. These needs mainly concerned the information to be presented, as well as to whom, when, for what type of patient, and how the information would be presented. The list of these needs led to the formulation of specifications for the PPT graphical user interface (GUI).

Early mock-ups based on these specifications were developed by a human factors specialist using Axure (Axure). These mock-ups represented static screenshots of the whole PPT GUI and used interface components and mock but realistic patient data to look as much like a real interface as possible and to present a realistic occupancy of the PED.

The mock-ups were presented to a focus group comprising 3 physicians, 2 registered nurses, 2 human factors specialists, and 2 software engineers. The final set of functional specifications as revised by the focus group was used to improve the mock PPT.

### Evaluation of the PPT’s Usability and Sorting Rules

The usability of the mock PPT and the relevance of the sorting rules integrated into the PPA were tested during a user testing session with 12 volunteers, in accordance with the recommendations for formative evaluations [41,42] (7 registered nurses and 5 physicians, none of whom took part to the work system analysis). User testing is a method for evaluating a product by directly observing the way users use the product. It makes it possible to identify the difficulties encountered by the users and the origin of the problems in the product [43].

**Figure 2:**
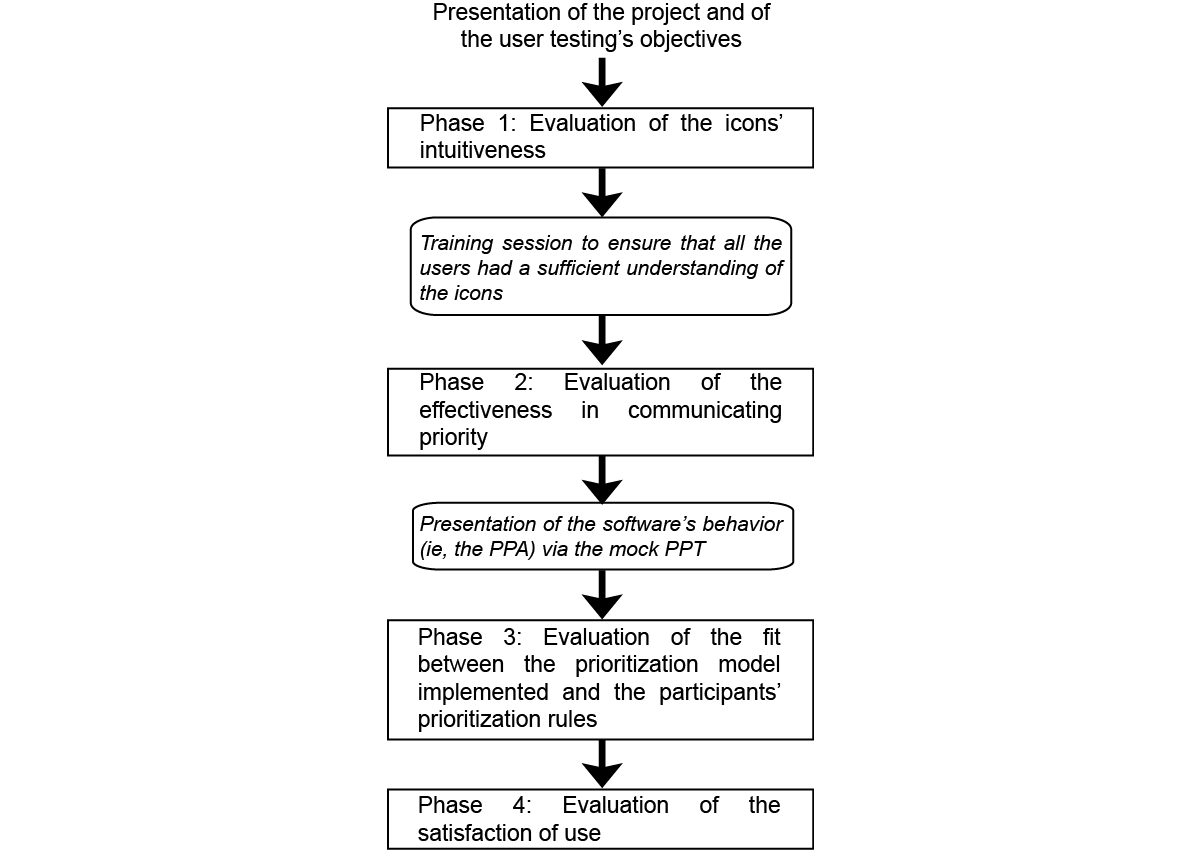
Rollout of the user testing. Testing sessions are presented in the straight-lined boxes and training sessions are presented in italic font. PPA: patient prioritization algorithm; PPT: patient prioritization tool.

A test session was divided into 4 phases alternating testing and training sessions (Figure 2).

Phase 1 tested the intuitiveness of the icons used to characterize the status of each patient. Participants were shown a mock-up of the tool that displayed 10 patients and were asked to explain how they interpreted each icon and each patient’s status. At the end, participants were given a training session on the icons so that they could perform phase 2.

Phase 2 tested the effectiveness in communicating priority. Participants were shown the same mock-up, in which 2 screenshots differed regarding the presence or absence of new patients and the current level of overcrowding. For each screenshot, participants were asked to identify the patient to whom they should first attend and to justify this decision. At the end, participants were shown a presentation of the system’s behavior to ensure that they had enough knowledge to perform phase 3.

Phase 3 tested the fit between the prioritization model implemented (the PPA) and the participants’ prioritization rules. This phase was inspired by the model-in-the-loop testing paradigm, a technique that simulates a model using an abstraction (eg, illustrations, text) to evaluate the behavior of that model [44,45]. This allows the model to be evaluated earlier in the design process with end users who are not experts in modeling and programming. For this phase, we created a simulator based on successive PPT screenshots to emulate the patient’s progress through the PED. This simulator presented 5 different patient scenarios covering all sorting rules integrated into the PPA. At certain points, the simulator was paused, and the participant was asked (1) to state what the next step in the PED process would be for the patient and (2) to place the patient at the corresponding location on the GUI.

In phase 4, we assessed the satisfaction of use and the perceived utility of the PPT. Participants were asked to fill out a French-language version of the System Usability Scale (SUS) [46] and to give their opinion of the PPT and on the prioritization rules implemented.

Data collected during the 4 phases were analyzed as follows.

In phase 1, to evaluate the intuitiveness of the icons, we calculated the proportion of participants that correctly interpreted each icon. In the event of misinterpretation, we sought to understand the reasons for poor intuitiveness of the design by qualitatively analyzing participants’ verbal statements.

In phase 2, effectiveness in communicating priority was analyzed. For each screenshot, we compared the participants’ choice of the top-priority patient with the patient indicated as such by the PPA. We sought to understand problems by analyzing participants’ verbal statements. If clinicians' prioritization and their justification for this decision were consistent with the organization proposed by the GUI, we presumed that the organization matched their work habits.

In phase 3, the fit between the prioritization model implemented (the PPA) and the participants’ prioritization rules was assessed by rating participants’ decisions on the patient’s position on the GUI as correct or incorrect compared with the patient’s position according to the PPA. In the event of discrepancies between participants’ choices and the application of the PPA, we analyzed verbal statements.

In phase 4, to assess satisfaction of use and perceived utility, the SUS score of all participants was averaged and compared with the standard established by Bangor et al [47]. A content analysis of the participants' verbalizations was carried out to identify the perceived advantages, drawbacks, and limitations of the PPT and PPA by the participants.

### Compliance With Ethical Standards

All procedures performed in studies involving human participants were in accordance with the French ethical standards and with the 1964 Declaration of Helsinki and its later amendments, or comparable ethical standards. Informed consent was obtained from all individual participants included in the study.

## Results

### Work System Analysis and the Tool’s Functional Specifications and Design

#### Work System Analysis

**Figure 3:**
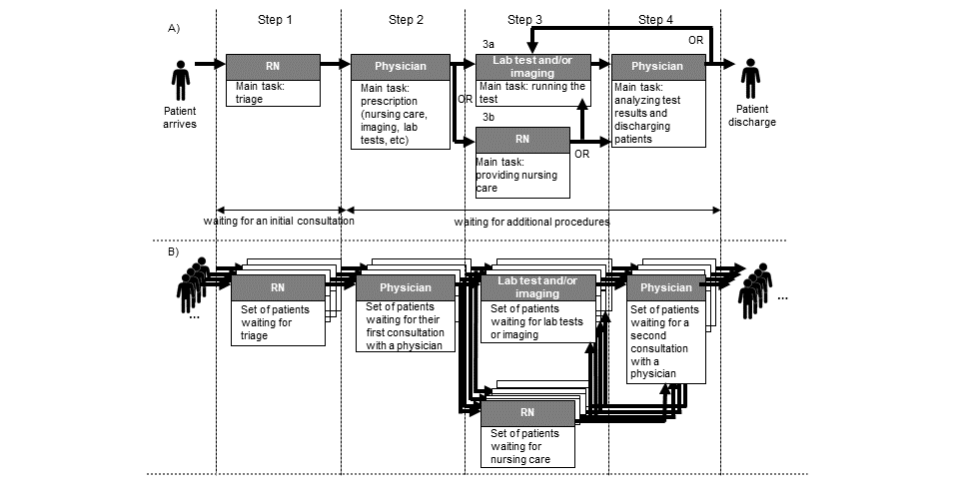
Schematic description of patients’ progression through the pediatric emergency department. Panel A describes the main tasks to be performed by the registered nurses and the physician at each step in the care process for a single patient. Panel B highlights that the pediatric emergency department care process is the same for all patients. RN: registered nurse.

A total of 1264 actions and 43 prioritization decisions were observed during the shadowing sessions (total of 27 hours). The care process is organized into 4 main steps regardless of the PED’s workload (Figure 3). Upon arrival in the PED, the patient is evaluated by a registered nurse (step 1), who determines the corresponding triage status. The patient then enters the care process. First, the patient sees a physician (step 2), who makes an initial diagnosis and prescribes the necessary lab tests, imaging, or nursing care. Next, the patient undergoes the prescribed lab tests or radiological examinations (step 3a) or nursing care (step 3b). When the lab test and imaging results are available or nursing care has been completed, the patient sees the physician again (step 4); the physician may prescribe further treatment or authorize the patient’s discharge. Throughout the patient flow process, physicians enter data into ResUrgences and complete the patient’s records (patient status, prescriptions, lab test results, notes, etc).

**Figure 4:**
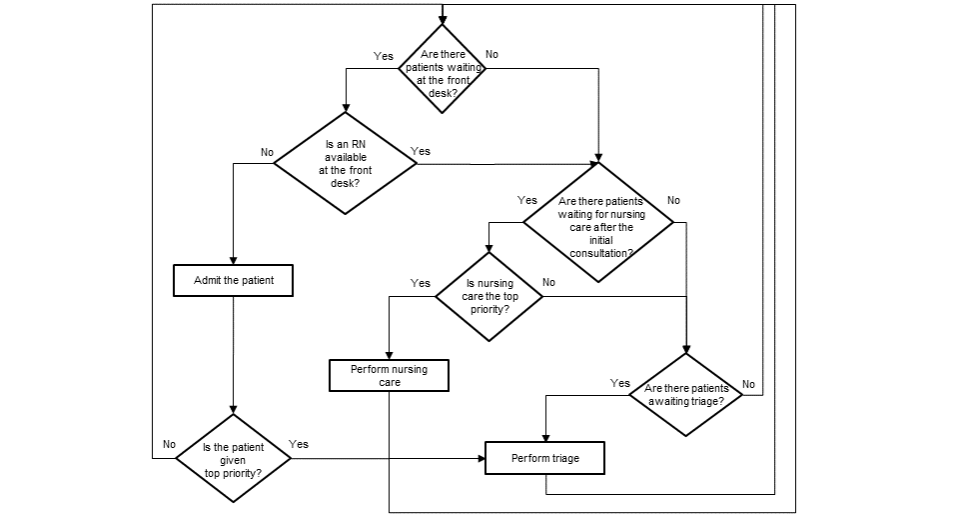
Example of RNs’ sorting rules. Actions are presented in the rectangular boxes and conditions for decision in the diamond-shaped boxes. RN: registered nurse.

To streamline patient flow through the PED, registered nurses and physicians apply various rules to prioritize patients to be attended. The main data used by clinicians to apply those rules are depicted in Textbox 1. Figure 4 provides an example of the sorting rules applied by registered nurses (Multimedia Appendix 1 for physicians).

**Main data used by the clinicians to manage patient flow during busy periods.**

**Patient’s information**

- Name
- Age
- Reason for admission
- Triage number

**Patient’s current position in the care process**

- Treatment by a registered nurse or physician
- Waiting time for further examinations
- Patient’s overall length of stay in the pediatric emergency department

**Patient tests**

- Tests prescribed
- Tests to be completed

Our observations of the timeliness of ResUrgences data entry showed that the data were representative of PED activity, even during busy periods. The median time between the receipt of data by the physicians and their data entry into ResUrgences was 136 seconds (IQR 67-345 seconds). During busy periods, even data that were first collected on paper were entered into ResUrgences no more than 2 minutes later (for details, see Schiro et al [48]). These results showed that ResUrgences data could be used to automatically feed the PPT and the PPA and inform clinicians on the progress of the patient through the care process.

#### Requirements and Specifications

The work system analysis enabled us to identify requirements for the PPT; it must (1) meet the needs of both physicians and registered nurses, (2) show physicians and registered nurses how the patients are distributed across the steps in the care process, and (3) display the current caseload and the corresponding priority levels.

Discussions during the focus group provided a consensus on more detailed specifications. First, patients in a life-threatening situation must not be affected by the algorithm because they are always treated with the highest level of priority. Second, the PPT should sort waiting patients according to whether they must see a nurse, see a physician, or undergo lab tests or imaging. Third, the PPT should prioritize patients to be seen by the registered nurses and the physicians. Patients waiting for lab tests or imaging results should be sorted as a function of their waiting time. Fourth, the tool should be fed directly with ResUrgences data so that clinicians do not have to enter the same data twice.

#### Design of the Mock PPT

**Figure 5:**
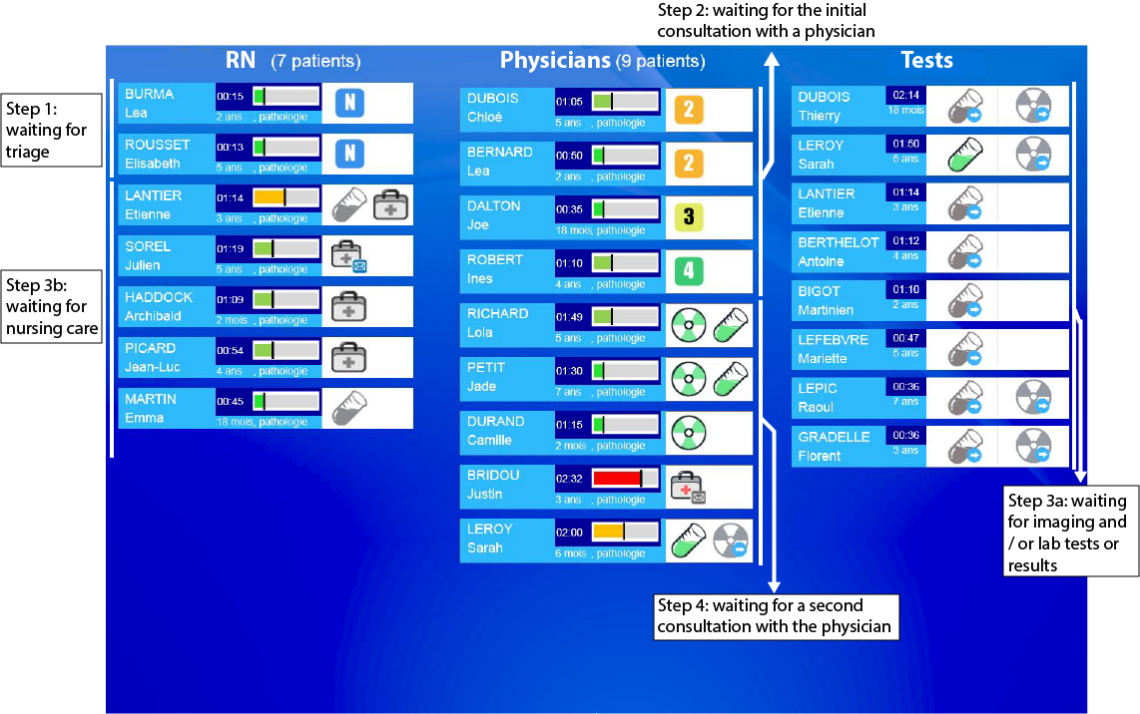
Mock-up patient prioritization tool’s main screen, which gives an overview of the patients and information on each patient. RN: registered nurse.

To meet the requirements, the mock PPT gives the status of patients progressing through the PED, along with an overview of all the patients in the PED (Figure 5). Each strip represents the patient, as well as the patient’s age, waiting time, reason for emergency (represented in the mock PPT by “pathology”), in-progress and pending cares/acts, triage number, and a delay indicator that compares the patient’s waiting time with the average waiting time of Lille University Hospital’s patients with a comparable reason for emergency (green ribbon when the patient's waiting time is less than the average waiting time, orange or red otherwise).

**Figure 6:**
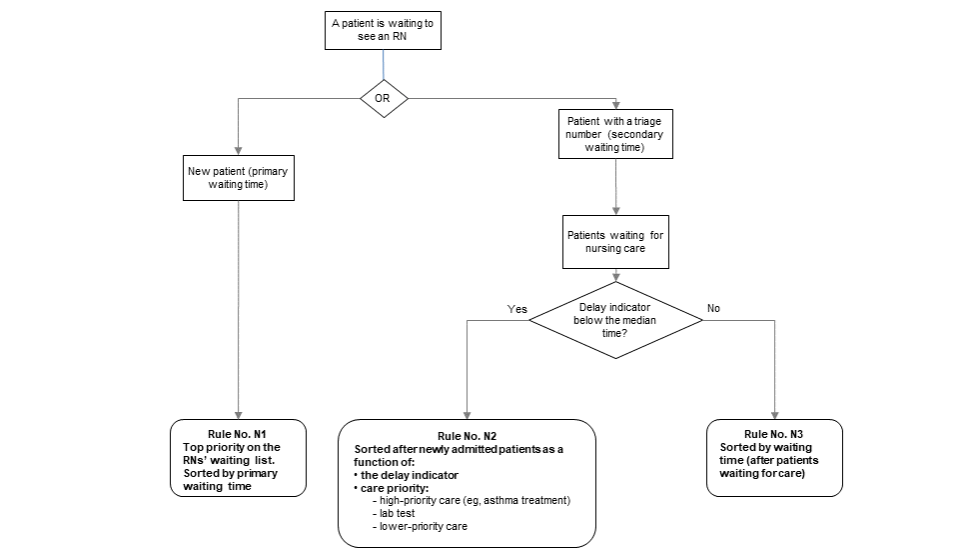
Sorting rules for the registered nurses, as integrated into the patient prioritization algorithm. Patients with life-threatening medical emergencies are always considered the highest priority and therefore do not appear in these sorting rules. RN: registered nurse.

Based on the staff’s strategies to prioritize patients, a set of sorting rules was developed for the registered nurses (Figure 6) and the physicians (Multimedia Appendix 2) and then aggregated into a PPA. This algorithm is automatically fed with data from ResUrgences. It calculates the status of each patient in real time and moves the patient’s strip through the blocks on the interface, which represent the main steps of the emergency care process, and through the lines within the blocks, suggesting the level of priority.

### Evaluation of the PPT’s Usability and Sorting Rules: User Testing

#### Phase 1: Intuitiveness of the Icons

**Figure 7:**
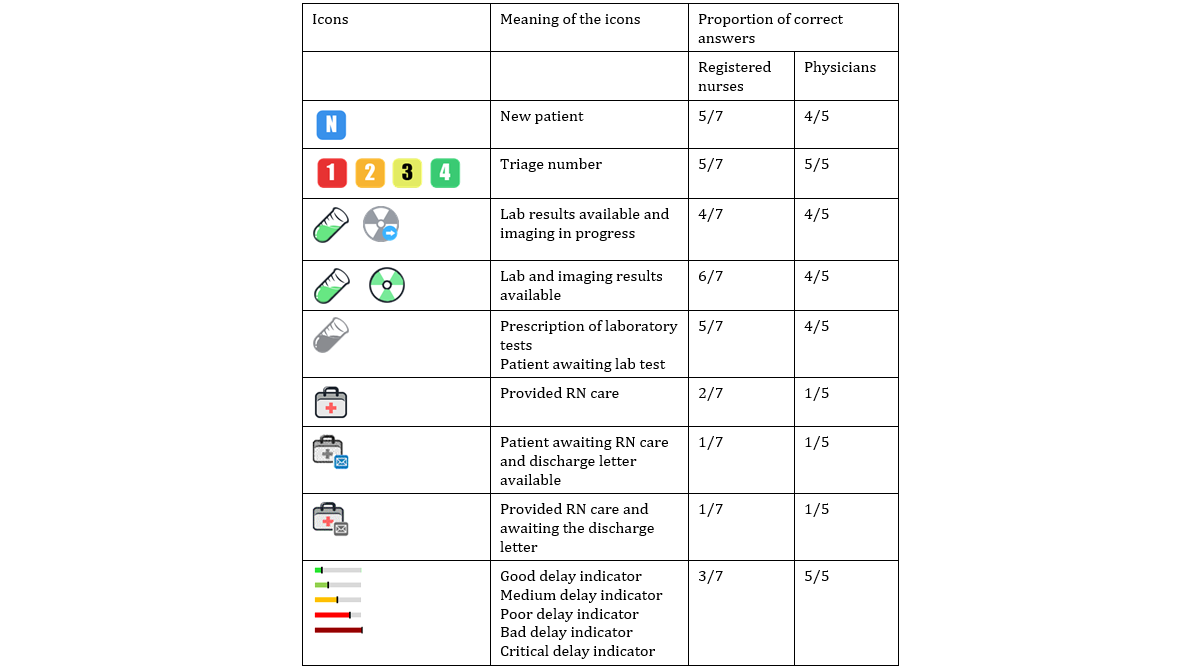
Results of phase 1 of the user testing: proportion of correct interpretation for each icon according to the profile of the participant. RN: registered nurse.

Most of the icons were interpreted correctly (Figure 7). Only icons depicting a doctor’s bag were not well interpreted, as only 2 of the 7 registered nurses and 1 of the 5 physicians interpreted them correctly. An analysis of the verbal statements showed that the participants either did not understand the icon’s meaning at all or thought it represented the patient’s records or a consultation with a specialist. Furthermore, the color coding (gray for “to do” and a color for “done”) was more easily understood for lab tests or imaging (5/7 registered nurses and 4/5 physicians) than for care provision (2/7 registered nurses and 1/5 physicians). The delay indicator was properly understood by all physicians but only by 3 of the 7 registered nurses. In fact, the registered nurses tended to interpret the delay indicator solely as a measure of the time elapsed since the patient's arrival in the PED (Table 1, quote 3). Physicians valued the delay indicator because it removed the need to schedule a discharge time, which would have constituted a source of stress (Table 1, quote 1).

**Table 1:** Quotes from registered nurses and physicians during phase 1 of the user testing.

Participant	Quote
Physician No. 1	“It’s good not to have an estimated discharge time, which is what I feared with this project. With the color coding, it’s easier to understand.”
Physician No. 2	“The ‘lab’ icon is easy to recognize, and the color change to indicate that the result is available is clear. But what happens when the result has been read and interpreted by the doctor, does it change? because this is another step in the care process.”
Registered nurse No. 3	“I did not understand the delay indicator immediately, but actually it's not bad – it’s important to help us know who to see first.”
Registered nurse No. 5	“The delay indicator is very interesting. You have to bear in mind that it’s not just the time.”

#### Phase 2: Effectiveness in Communicating Priority

For the view with new patients, 6 of the 7 registered nurses and all physicians understood that the high-priority patients were those at the top of their respective column. However, 2 registered nurses considered that the choice also depended on the patient’s health status (Table 2, quote 4). For the view with no new patients, all registered nurses and 4 out of 5 physicians agreed with the GUI’s organizational structure. The only inconsistent answer was related to the application of a different strategy by a physician (Table 2, quote 1).

**Table 2:** Quotes from registered nurses and physicians during phase 2 of the user testing.

Participant	Quote
Physician No. 4	“I’d first see the nurse and tell her to take care of the patients at the top of the list, so that they can get discharged.”
Physician No. 5	“Sometimes there are patients who are still under our responsibility but for whom there is no longer anything urgent because they are just waiting to be discharged; it is not the same as waiting to see the doctor for a diagnosis.”
Registered nurse No. 1	“For some decisions, it's going to depend on how busy the ward is, how many patients there are.”
Registered nurse No. 2	“It depends on the severity of the new patients’ status. If I see that a new arrival has a minor injury, I’ll do a blood test for another patient first because I know that it’ll take a while to get the results.”

Overall, our analysis of the participants' verbal statements did not identify any difficulties in understanding how the information was organized or how the patients were located in the GUI. A nurse did indicate that it was necessary to provide a view of the department's occupancy to help him make certain care decisions (Table 2, quote 3). A doctor pointed out that, in the doctor column, two types of patients were mixed: those waiting for an auscultation or a diagnosis and those waiting to be discharged. However, the urgency is not the same for these two types (Table 2, quote 2).

#### Phase 3: Fit Between the Prioritization Model Implemented (the PPA) and the Participants’ Prioritization Rules

Overall, participants tended to agree with the PPA’s decisions—they placed the patients in the expected column with the expected priority. Proportions of correct decisions were 87% (61/70) for registered nurses and 98% (49/50) for physicians (Table 3). Overall, the PPA was validated by most of the users.

**Table 3:** Results of phase 3 of the user testing: proportion of correct prediction according to the patient case and the profile of the participant, along with explanations in case of erroneous prediction.

Scenarios		Physicians’ answers			Registered nurses’ answers		
Patient case	Rule	Column, n/N	Block/line, n/N	Explanation	Column, n/N	Block/line, n/N	Explanation
Case 1	Rule No. Ph3	5/5	4/5	Patients do not always progress to the next step when lab results are available (n=1)	7/7	4/7	Patient not prioritized (no block or line) (n=2) or without the expected icon (n=1)
Case 2	Rule No. Ph1	5/5	5/5	N/A^a^	7/7	6/7	Patient not prioritized because the RN^b^ needs to know the type of pathology to place the patient (n=1)
Case 3	Rule No. N3	5/5	5/5	N/A	6/7	6/7	One RN failed to understand that, after discharge, the patient had to be moved outside the interface (n=1)
Case 4	Rule No. N2 Rule No. Ph4 Rule No. Ph5 Rule No. Ph6	5/5	5/5	N/A	6/7	5/7	One RN did not move the patient to another column (n=1) Another relied exclusively on the delay indicator and opted for the wrong block (n=1)
Case 5	Rule No. N1	5/5	5/5	N/A	7/7	7/7	N/A

^a^N/A: not applicable.

^b^RN: registered nurse.

Because of the use of the term “pathology” instead of an actual reason for emergency on the mock-up, some clinicians found it difficult to place the patients as presented in the patient strip. They also pointed out a limitation concerning the time elapsed between the availability of test/imaging results and the moment of their interpretation. In fact, lab results seldom arrive simultaneously; in some situations, the physician checks that all the results are available before seeing the patient or deciding about discharge (Table 4, quote 1). However, the PPA only accesses the availability of the lab and imaging results to calculate the new position of the patient. This may lead to discrepancies between the actual step in the care process that the patient is in and the displayed step.

A few participants also criticized that the PPA did not include subjective elements that may enter into clinicians’ decision for prioritizing patients (Table 4, quote 3).

**Table 4:** Quotes from registered nurses and physicians during phases 3 and 4 of the user testing.

Participant	Quote
Physician No. 2	“There’s a missing step here: the physician might read the lab results but not do anything [because some lab results are still missing]; the ‘R’ [indicating that the lab results are available] disappears from ResUrgences because the results have been accepted and the patient has been seen by the physician, but nothing happens and s/he does not move through to the next step.”
Physician No. 3	“This appears to be quite useful. It would be good to have screens in the [emergency department]. We’ll need access to the computer, as with ResUrgences. Then, I can help out even if I’m not caring for a patient...because sometimes ResUrgences shows you that there are lots of people waiting but that’s not the reason why the [emergency department] is disorganized.”
Physician No. 5	“I’m not sure whether we need to base our actions on that or not, because we use subjective criteria that cannot be taken into account. However, the system has already done a huge amount of work in organizing the patients!”
Registered nurse No. 1	“I think it’s a good idea. I will go see patients at the top of the list in ResUrgences. That will help me to avoid consulting them one by one...”
Registered nurse No. 7	“I’ll place more trust in what I’m told [by my colleagues] than in a tool but this is a good add-on.”

#### Phase 4: Satisfaction of Use and Perceived Utility

The average SUS score was 70 (on a scale ranging from 0 to 100), which highlighted a good satisfaction [47]. Overall, the PPT was perceived as being helpful for prioritizing patients (eg, Table 4, quote 2). However, participants said that they would continue to consult ResUrgences in addition to the PPT to organize their work (Table 4, quote 4). Physicians found the PPT very useful as an overview of the department’s activity and, at the same time, an indication of work that they must do immediately (Table 4, quote 2). Registered nurses found it useful too and stated that it would save time when compared with using ResUrgences alone (Table 4, quote 4).

## Discussion

### Principal Results

This case study aimed to present the human-centered design of a PPT and of the PPA it relies on to show how to apply human-centered design methods to the design of a prioritization system. Representative end users were involved early in the design process. We performed a work system analysis and, based on the specifications ensuing from it, we designed a PPT along with a PPA. Finally, we performed user testing on mock-ups simulating how the PPT and PPA work.

Results of the work system analysis underpinned the entire design process, from specification of the users’ needs to the development of the PPA. The work system analysis enabled us to understand how the PED was organized and how clinicians managed the patient flow. This analysis also showed a short data entry time in ResUrgences, which would indicate that these data represent the patient's management in real time and therefore can be used to automatically populate the PPA and PPT. Furthermore, this analysis provided specifications needed to design the tool. Lastly, the work system analysis enabled us to build scenarios for designing the PPA and the PPT’s GUI and helped us design the evaluation plan.

**Figure 8:**
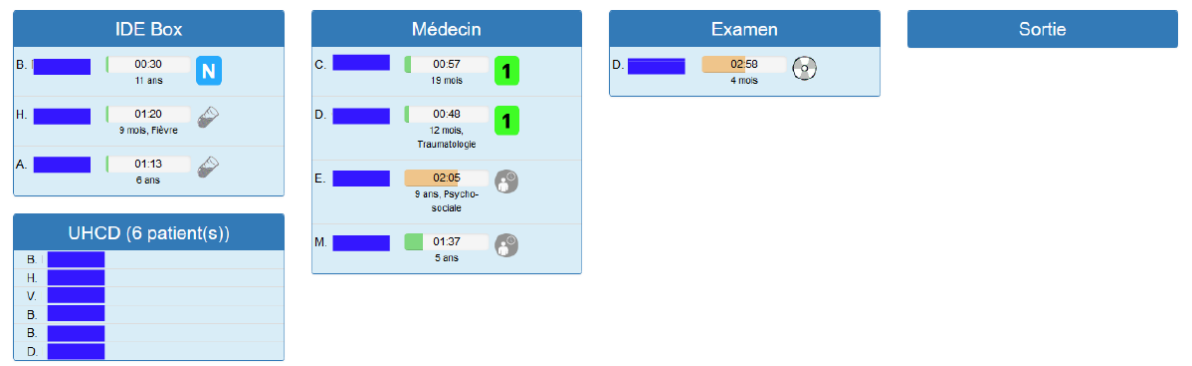
Screenshot of the prototype patient prioritization tool after re-engineering (blue rectangles hide patient identity).

In the user testing, we simulated how the PPT and the PPA would work by animating successive screens of the mock-up, populated with fake but realistic sets of ResUrgences data. Applying an adapted model-in-the-loop testing paradigm [44,45] during the early steps of the design process enabled us to obtain feedback on the GUI before developing the prototype PPT and to ensure that health care professionals understood the PPA they evaluated. The results of the user testing were used to make decisions to improve the PPT and the PPA. Figure 8 represents the new version of the PPT.

Most of the changes concern the icons and the GUI’s organization. For example, the doctor’s bag icon had several meanings depending on its color and its combination with another symbol (“nursing care” or “discharge letter available”). It was either not understood or was mistaken for a representation of the patient’s records or a consultation with a specialist. In order to avoid this polysemy, it is no longer associated with the discharge letter and represents only the realization of nursing care. In addition, the delay indicator was misinterpreted by nurses because this type of indicator is not usual and not present in other software. Explanations of the calculation of this indicator and its meaning were given to users during training sessions, as well as in posters displayed next to the PPT screen in the department. In regard to the GUI, the time of patient presence in the ward was integrated in the center of the progress indicator (instead of to the left) to show that it was associated with the calculation of the indicator. Another issue was that the mock-up presented only patients present in the emergency department care and consultation sector and excluded the emergency department short-stay hospitalization sector. However, for some decisions, clinicians need to know the occupancy rate of the entire department. To provide clinicians with access to this information, a box summarizing the number of patients in the hospitalization sector was added. Finally, patients waiting for a doctor to sign their discharge letter were previously mixed in with patients waiting for a consultation. However, from a physician work organization point of view, having a lot of patients waiting for their discharge letter does not have the same consequences as having a lot of patients waiting for a diagnosis. The doctor can quickly release several patients by signing the discharge letters one after the other. Therefore, a fourth block, “Discharge,” was added to the GUI for patients who are waiting for their discharge letter to leave the PED. The addition of this block allowed us to eliminate the icons representing that a patient is waiting for discharge letter.

A number of sorting rules in the PPA were also modified or created. For instance, rules were changed to enable a distinction between the test and imaging results that were available in ResUrgences and those that had been interpreted by the doctor, because these represent different steps of patient care.

Overall, this human-centered design methodology was useful to design a PPT that complies with clinicians’ workflow and that automatically retrieves data from the patient management software.

Taking account of end users’ feedback early in the design process helped deliver solid specifications to the developers and enabled us to develop the prototype PPT very quickly (10 person-months, including integration with ResUrgences). The PPT prototype has been deployed in Lille University Hospital’s PED. Four PPT screens were implemented, 2 in the physicians’ rooms (main office and residents’ office) and 2 in nursing rooms, each time right next to the ResUrgences screen that summarizes the PED’s patient information but that does not prioritize the patients or organize their care accordingly.

This way, when watching ResUrgences, clinicians can quickly access patient prioritization suggestions on the adjacent screen without having to reenter data. Until now, clinicians had to search for information about patients, such as their reason for entry and their waiting time, and then compare them to decide who to take care of first. Now this cognitive effort should be alleviated by the PPT's prioritization suggestions, which are based on decision trees that clinicians were implementing. Using the PPT may help physicians and nurses have a better awareness of the PED crowding and help them improve the management of the department’s resources and beds. Consequently, this tool can help reduce patients’ waiting time, especially for critical patients and before time-sensitive treatments and procedures.

### Limitations

This case study presents the first iterations of a human-centered design of a PPT and PPA. A formative evaluation by user testing was conducted and the results were used to modify the GUI and the PPA. In a conventional human-centered design, a summative evaluation would have been conducted to ensure that the usability of the PPT and PPA had been improved and that there were no residual issues that could impede use or generate adverse events. However, clinicians expressed a desire to see the tool installed quickly in the PED. With respect to the intent of use of the tool (to help prioritize patients, excluding patients in a life-threatening situation, without imposing this prioritization), the potential risks arising from usability issues would be misinterpretations of the information provided, with the worst consequence being a possible increase in waiting times for some patients and a rejection of the tool by clinicians. These risks were deemed acceptable and, in agreement with the department head, the tool was installed while ensuring a support and monitoring program to continuously evaluate its use and usability. During presentations of the tool and observations about its use at the time of its installation and during ongoing studies, the impressions and comments of clinicians were collected and analyzed. This feedback did not identify any usability issues that hindered users; they helped us clarify the interface further (eg, with the addition of a “pending decision” icon to inform users that the patient is waiting for a specialist's opinion or of a “homecoming” icon to distinguish between patients who are discharged and returned home and patients who are discharged but waiting for hospitalization). Even after the installation of the PPT prototype, end users remain at the heart of the design and evaluation process to ensure the PPT fits their needs and is useful.

A second limit relates to the designed tool. Its design was based on data that could be retrieved from ResUrgences and used by clinicians to prioritize patients. However, some marginally used elements are not entered into ResUrgences. For example, emotional elements such as crying infants can sometimes prompt clinicians to see a patient more quickly when this is not to the detriment of other patients. Because they are not entered into ResUrgences, these emotional factors cannot be taken into account when suggesting the next patients to see. Despite this technical limitation, the tool is useful because it provides all the other information clinicians need to get an idea of the next patients to see and suggestions for prioritization on the same interface. Clinicians are then free to consider other contextual elements when making their prioritization decision.

### Future Research

This study was the first step of the Optimum project. Now that the PPT and PPA have been developed and installed in Lille University Hospital's PED, studies are being carried out to evaluate the use and impact of this tool. A first study was conducted to ensure that the information displayed on the PPT screen correctly reflected the stage of care of the patient. Although there were a few discrepancies due to late entry of information, the distribution of patients in the different stages of care on the screen accurately reflected the actual distribution of patients [48]. Another research study is running to assess how clinicians are appropriating and using the PPT, how the tool is integrating their activity, and how it is satisfying their needs. This study is a prerequisite for investigating the impact of the usage of this tool on clinicians' work, and it raises future research questions: Has the use of the PPT changed their situation awareness and their cognitive load when choosing a patient? What is the impact of the use of the PPT on the prioritization of patients and ultimately on time-sensitive treatments and procedures for serious conditions?

Finally, the PPT and PPA were designed following a human-centered design, in which end users were the doctors and nurses of the PED of Lille University Hospital in France. The tool therefore integrates a work model as well as decision trees that correspond to those applied in the hospital's PED. Before this prioritization tool can be deployed in other EDs of Lille University Hospital (eg, general, ophthalmological, psychiatric, etc), it will be necessary to ensure that the workflow and prioritization rules are the same there; if not, then the PPT and PPA will have to be adapted to these new contexts. Similarly, transposing the PPT and PPA to other hospitals or other countries would first require analyzing future work contexts and adapting the PPT and PPA accordingly.

### Conclusion

This study details the integration of human factors into the design process for a PPT and PPA for a PED. A human-centered design allowed the needs of end users and their work constraints to be considered early in the design cycle. Workflow analysis allowed us to (1) identify the information needed for clinicians to prioritize patients, (2) model prioritization decisions in order to implement them as an algorithm in the PPT, and (3) verify that the information entered in the patient management software was entered quickly enough to represent the progression of patient management. A mock-up was developed based on the results of the workflow analysis. It was tested by user testing. Although some usability issues were identified, the majority of clinicians understood the GUI and the prioritization algorithm and felt that the tool could help them in their task. The results of the tests led to minor modifications to some elements of the GUI and the prioritization algorithm in order to improve the usability and usefulness of the PPT. A prototype version of the PPT has been developed and implemented in the PED.

Including end users throughout the design process through user-centered design helps guide the design and evaluation of health technologies so that they align as closely as possible to the reality of users’ needs and activities.

## Appendix

Multimedia Appendix 1 Example of physicians’ sorting rules. Actions are presented in the rectangle boxes, conditions for decision in the diamond-shaped boxes.

Multimedia Appendix 2 Sorting rules for the physicians as integrated into the PPA. Patients with a life-threatening medical emergency are always considered with the highest priority and therefore do not appear in those sorting rules.

## Abbreviations

ED: emergency department
GUI: graphical user interface
PED: pediatric emergency department
PPA: patient prioritization algorithm
PPT: patient prioritization tool
SUS: System Usability Scale
