# Supplementary figures and images for: Applying a Human-Centered Design to Develop a Patient Prioritization Tool for a Pediatric Emergency Department: Detailed Case Study of First Iterations
Source: JMIR Hum Factors. 2020 Sep 4;7(3):e18427. doi: 10.2196/18427 (PMC7501580; doi:10.2196/18427)

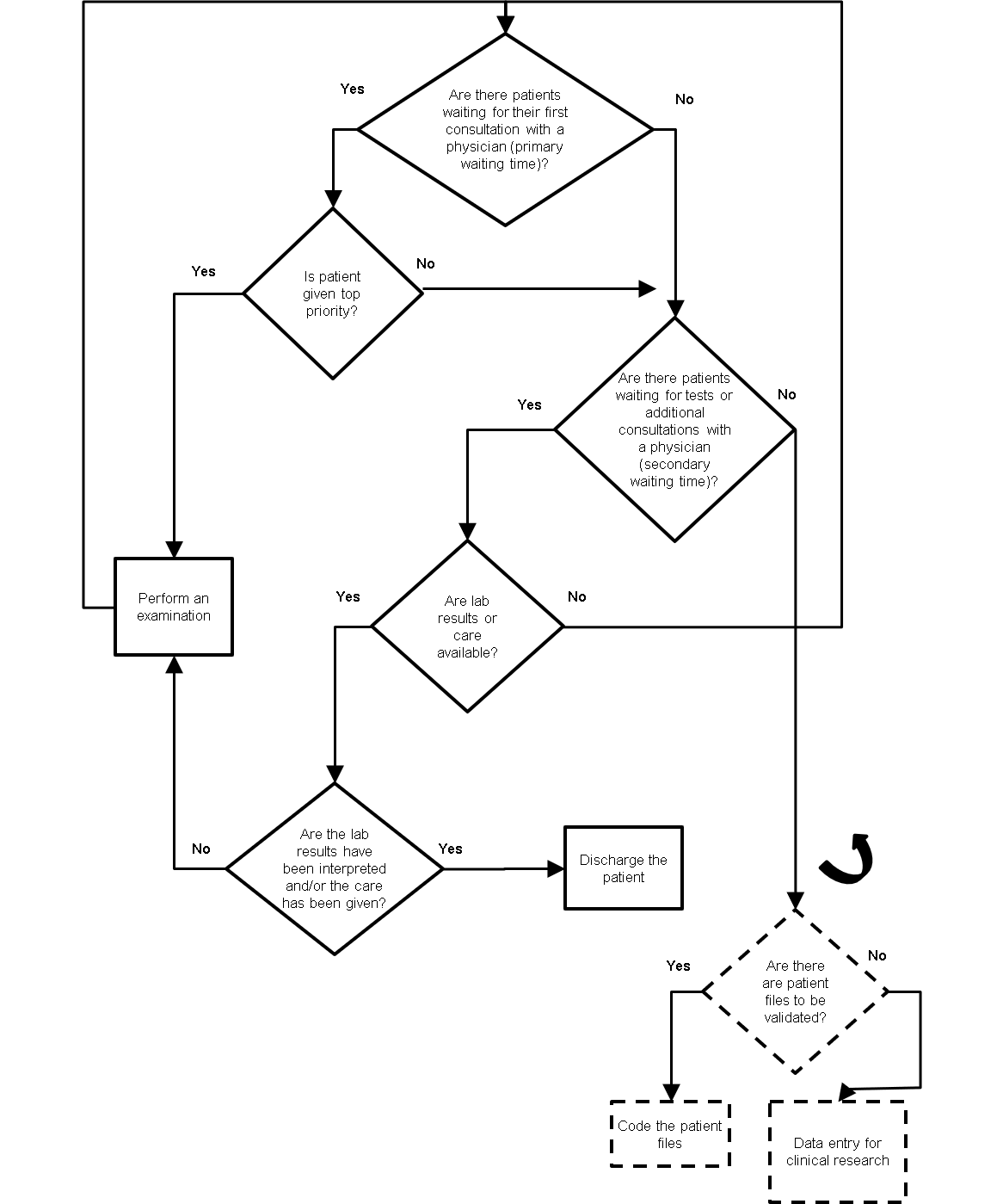

Supplement: Multimedia Appendix 1 [file humanfactors_v7i3e18427_app1.png]

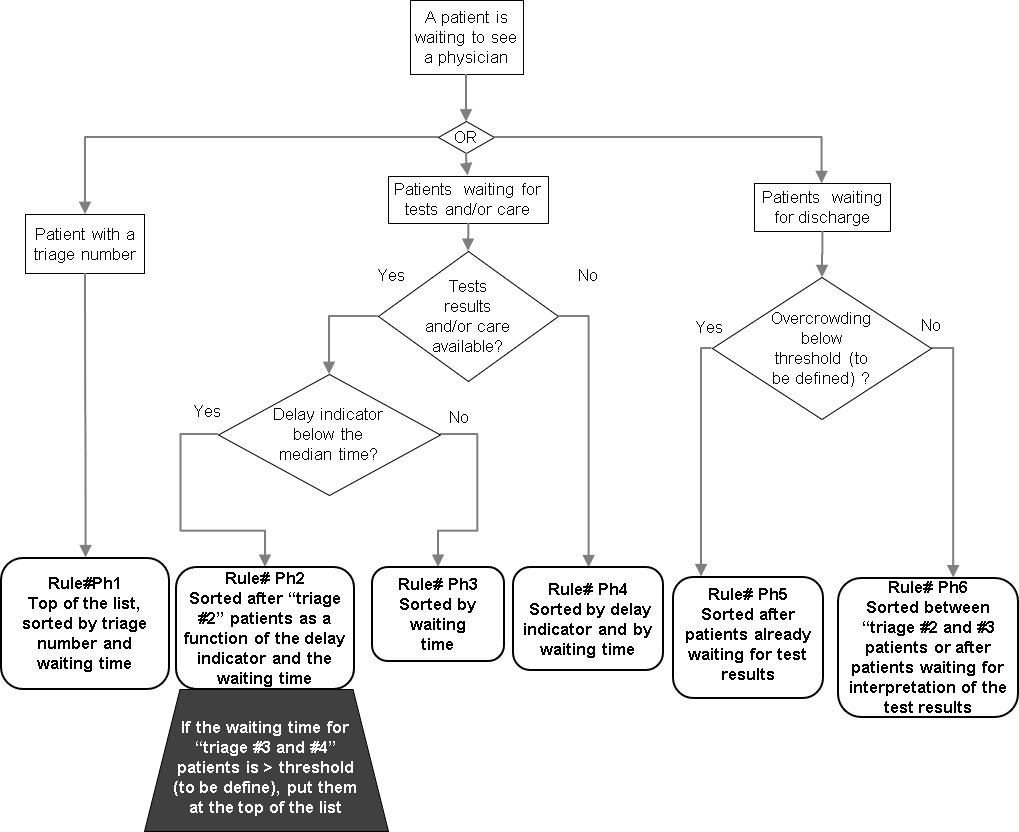

Supplement: Multimedia Appendix 2 [file humanfactors_v7i3e18427_app2.png]
